# Supplementary material for: 6mA-DNA-binding factor Jumu controls maternal-to-zygotic transition upstream of Zelda
Source: Nat Commun. 2019 May 17;10:2219. doi: 10.1038/s41467-019-10202-3 (PMC6525185; doi:10.1038/s41467-019-10202-3)
Supplement: Supplementary file 1 — Supplementary Information [file 41467_2019_10202_MOESM1_ESM.pdf]

Supplementary Information

6mA-DNA-binding factor Jumu controls maternal-to-zygotic transition by regulating Zelda

He, Zhang, Wang and Gao et al.

Supplementary Figure 1

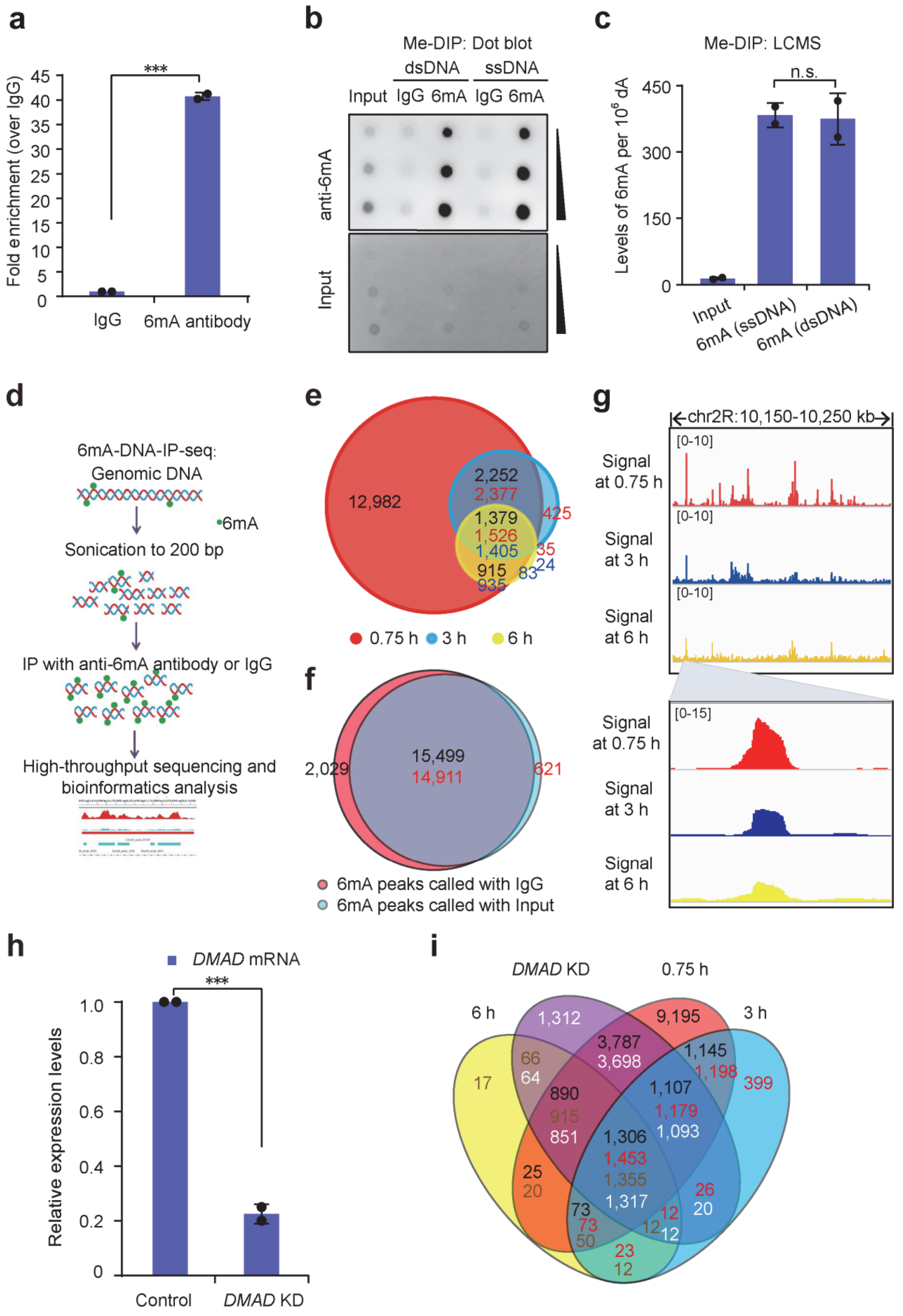

Supplementary Figure 1. Dynamic distribution of 6mA in *Drosophila* early embryo genomes.

(a) The methylated DNA fragments catalyzed by bacterial DAM methylase were used to measure affinity of the anti-6mA antibody (Abcam) in DNA-IP experiments. In this assay, IgG was used as control. The IPed products were then subjected to qPCR analysis. Data expressed as means of 2 independent experiments. Error bars indicate mean ± s.d. The two-tailed Student's t test was used to analyze statistical variance. ***, $P < 0.001$. **(b)** The immunoprecipitated DNA using both single-stranded DNA (ssDNA) and double-stranded DNA (dsDNA) was subjected to dot blot assays using anti-6mA antibody (Abcam). Methylene blue hydrate staining was performed to determine the relative levels of loaded DNA. **(c)** Abundance of 6mA in the immunoprecipitated DNA using both ssDNA and dsDNA fragments was measured by mass spectrometry. Data expressed as means of 2 independent experiments. Error bars indicate mean ± s.d. The two-tailed Student's t test was used to analyze statistical variance. n.s., not significant. **(d)** Schematic diagram of 6mA-DNA-IP-Seq. **(e)** Overlap of 6mA enrichment peaks in 0.75-, 3-, and 6-hour stage embryos. A peak at one stage may overlap with more than one peak at another stage. In Fig. 1b, numbers from only one stage was shown. In Supplementary Figure 1e, numbers corresponding to all three stages were displayed, and black for 0.75-hour, red for 3-hour and blue for 6-hour. **(f)** Overlap of 6mA peaks called by IgG as control and Input as control. Numbers of IgG-controlled 6mA peaks (black) and Input-controlled 6mA peaks (white) were displayed. **(g)** Examples of 6mA-marked regions in which the 6mA modification signals were dynamically changed. **(h)** DMAD was effectively knocked down by the *DMAD* double-stranded RNAs. Data expressed as means of 2 independent experiments. Error bars indicate mean ± s.d. The two-tailed Student's t test was used to analyze statistical variance. ***, $P < 0.001$. KD, knockdown. **(i)** Overlap of 6mA peaks identified in 0.75-, 3- and 6-hour of wild type and 6-hour *DMAD* knockdown (KD) samples. Source data are provided as a Source Data file.

Supplementary Figure 2

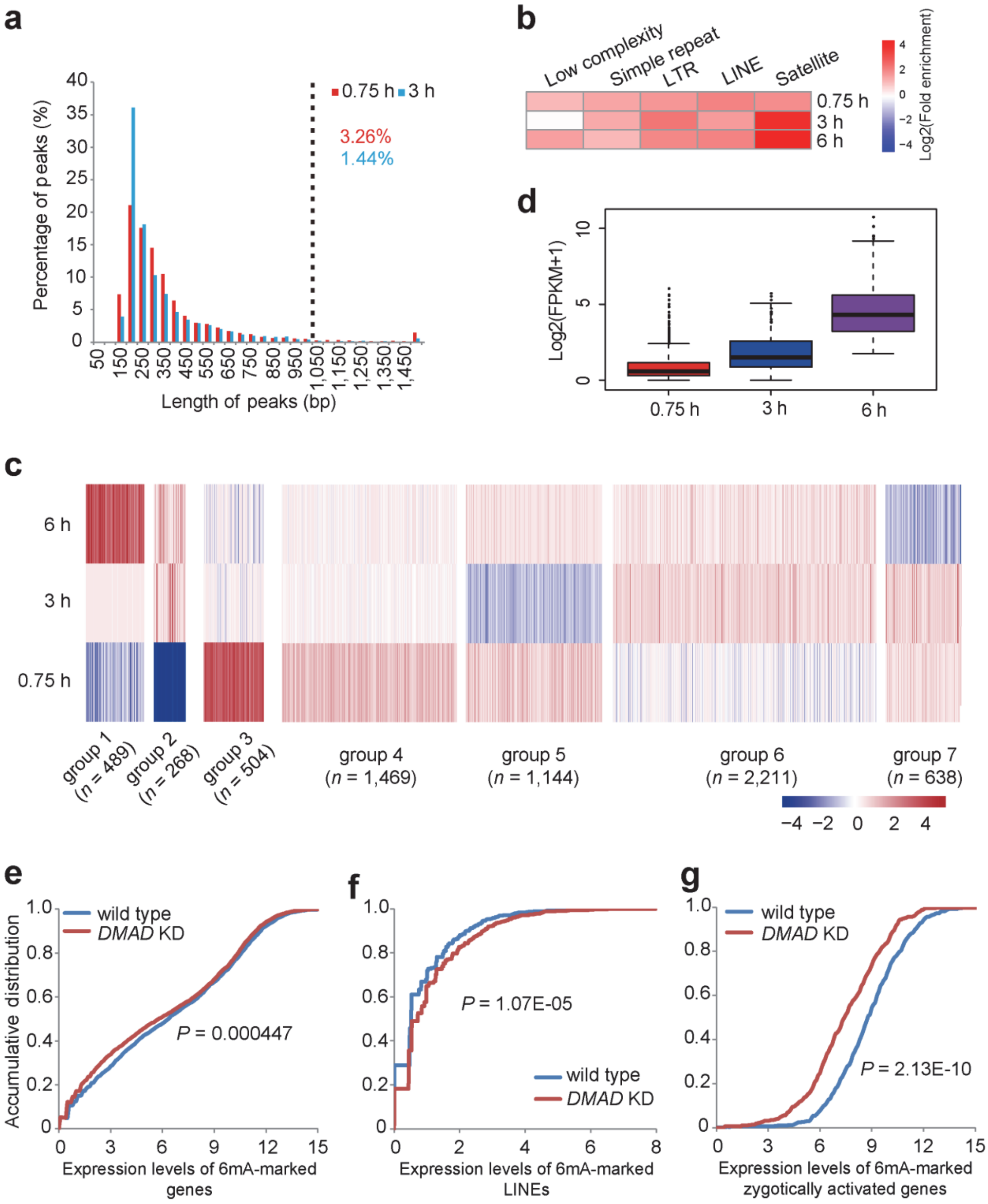

Supplementary Figure 2. 6mA marks coding genes and associates with gene expression in early embryos.

(a) The length distribution of peaks at 0.75-hour and 3-hour stages**. (b)** Comparison of observed versus expected distributions of 6mA modification and 6mA was enriched in multiple repeat elements. **(c)** Cluster analysis classified the 6,723 genes with significantly differential expression between at least two stages (at 0.75-, 3-, and 6-hour stages) into 7 groups. The colors in the heatmap display relative expression levels of genes at the three developmental stages. For each group, the number of genes was indicated. **(d)** The expression distribution of group 1 of genes in different stages. The expression level of genes was given in a log 2 scale [log2(FPKM+1)]. For boxplot, the centre line indicates the median, the bottom and top of the box show the first and third quartiles of the data, and the whiskers show the minimum and maximum values. **(e-g)** Accumulative distribution of expression levels of 6mA-marked genes (e), 6mA-marked LINEs (f), zygotically activated genes (g) in wild type and *DMAD* knockdown (KD) embryos. *P* values were calculated by two-tailed Student's t test. Source data are provided as a Source Data file.

Supplementary Figure 3

a

Mass Spec-identified 6mA-associating candidate proteins in early embryo				
Apt (CG5393)	Bab1(CG9097)	CG5098	CoRest(CG42687)	Dan(CG11849)
Dp(CG4654)	E2f2(CG1071)	Eip75B(CG8127)	Glu(CG11397)	Hsf(CG5748)
Jumu(CG4029)	L(3)neo38(CG6930)	Ocm(CG3363)	Org-1(CG11202)	Retn(CG5403)
Run(CG1849)	SA(CG3423)	Scm(CG9495)	Slp1(CG16738)	SMC2(CG10212)
Srp(CG3992)	Su(Hw)(CG8573)	Su(var)3-7(CG8599)	Su(z)12(CG8013)	Trl(CG33261)

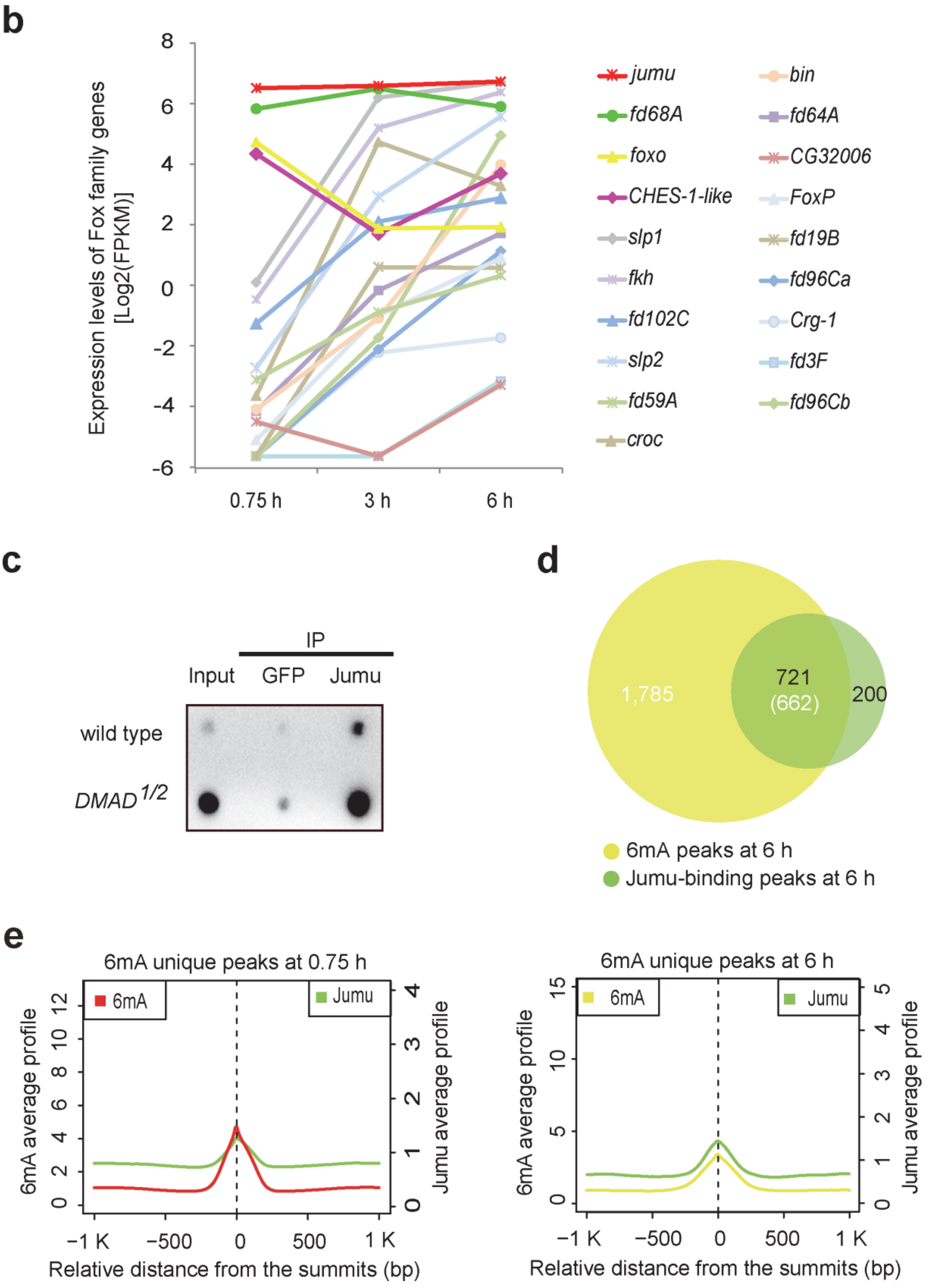

Supplementary Figure 3. Jumu is a 6mA-marked DNA-binding protein.

(a) Mass Spec-identified 6mA-associating candidate proteins in early embryos. **(b)** Expression levels of the Forkhead box family of transcription factors in 0.75-, 3-, and 6-hour stage embryos. **(c)** Purified Flag-Jumu or Flag-GFP (control) proteins were incubated with genomic DNA from wild type and *DMAD* mutant *Drosophila* brains. The DNA captured by anti-Flag beads was used in dot blot assays to assess the binding activity of Jumu protein with the 6mA-marked DNA. **(d)** Overlap of 6mA enrichment peaks and Jumu-binding peaks at 6-hour. **(e)** The average signal profile of 6mA and Jumu-binding in unique 6mA peaks of 0.75-hour and 6-hour. Source data are provided as a Source Data file.

Supplementary Figure 4

a

Genotype of embryos	Hatching rate		
	Replicate 1	Replicate 2	Replicate 3
Wild type	0.87 (710/816*)	0.91 (525/575*)	0.91 (530/585*)
jumu$^{(M-,Z+)}$ mutant	0.28 (114/402*)	0.28 (73/262*)	0.19 (150/775*)
jumu$^{(M-,Z-)}$ mutant	0.11 (51/455*)	0.15 (160/1,070*)	0.11 (46/409*)
jumu$^{(M+,Z+)}$	0.89 (405/455*)	0.90 (575/641*)	0.89 (325/365*)

b

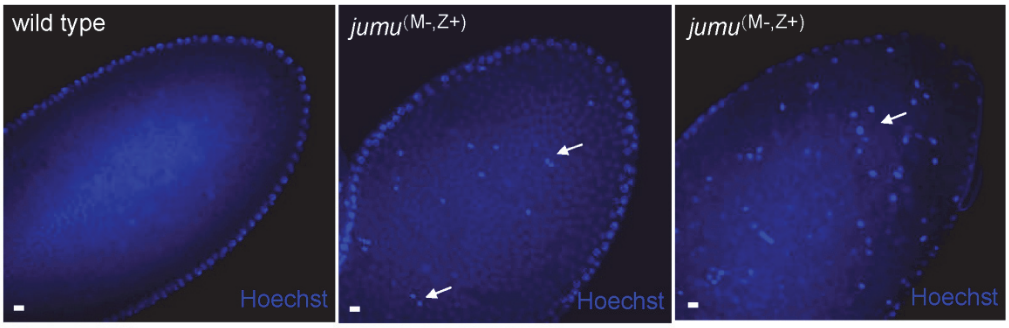

c

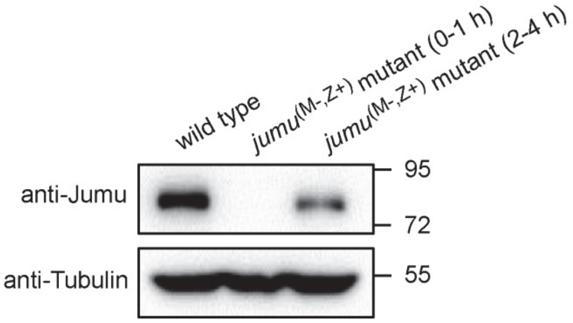

Supplementary Figure 4. Jumu functions as a maternal factor to regulate embryogenesis.

(a) Statistics of hatching rate of embryos with indicated genotypes. *, Sample size. **(b)** Nuclear fallout in embryos derived from wild type and maternal *jumu* mutant embryos at stage of 9-13 cycles. Scale bar, 10 µm. **(c)** Western blot experiments were performed to show that Jumu was zygotically expressed in *jumu*$^{(M-, Z+)}$ mutant embryos. Source data are provided as a Source Data file.

Supplementary Figure 5

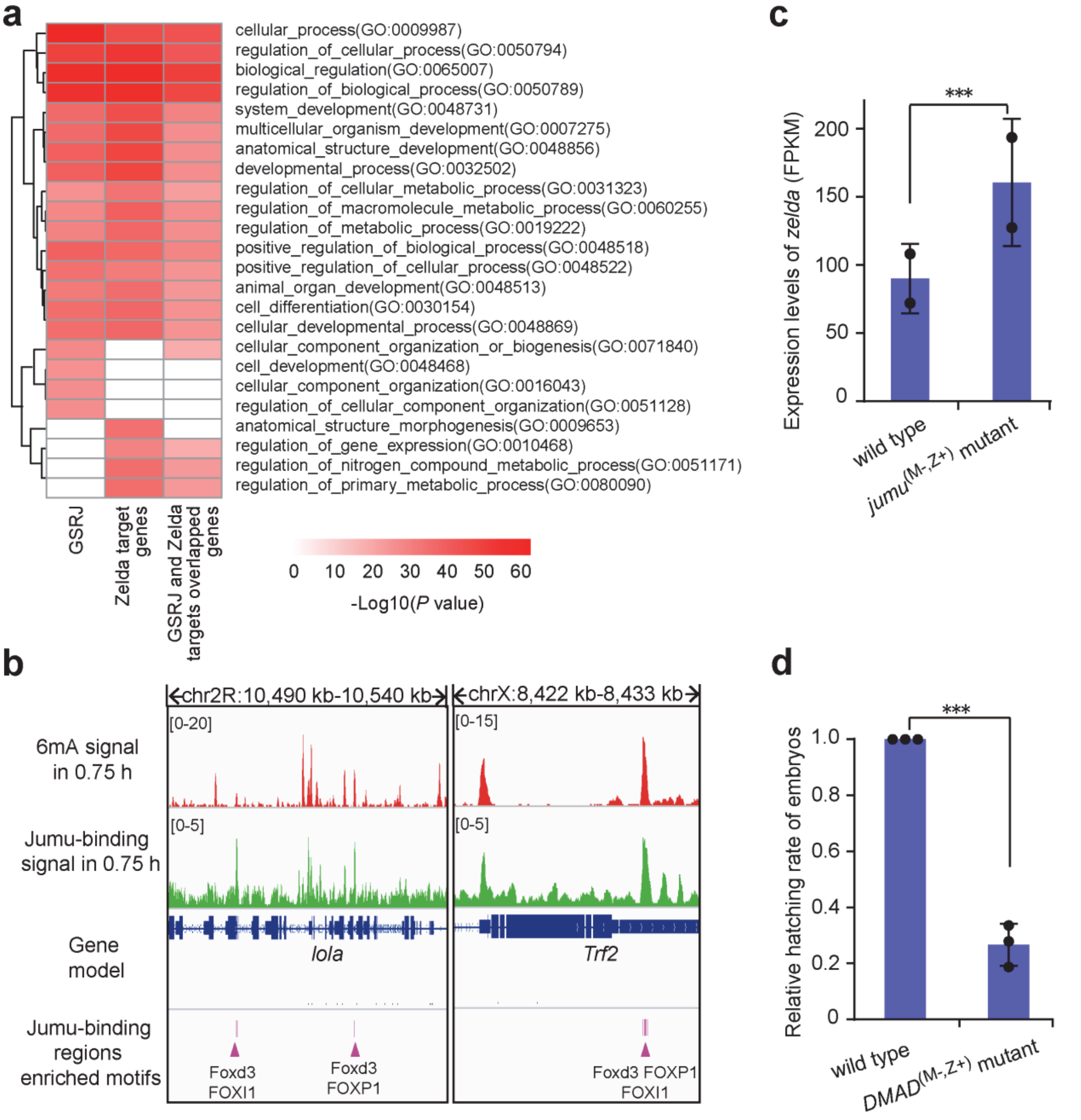

e

Genotype of embryos	Hatching rate		
	Replicate 1	Replicate 2	Replicate 3
Wild type	0.79 (165/209)	0.84 (768/914)	0.81 (199/245)
DMAD$^{(M-,Z+)}$ mutant	0.22 (4/18) 400*	0.16 (6/38) 791*	0.28 (9/32) 888*

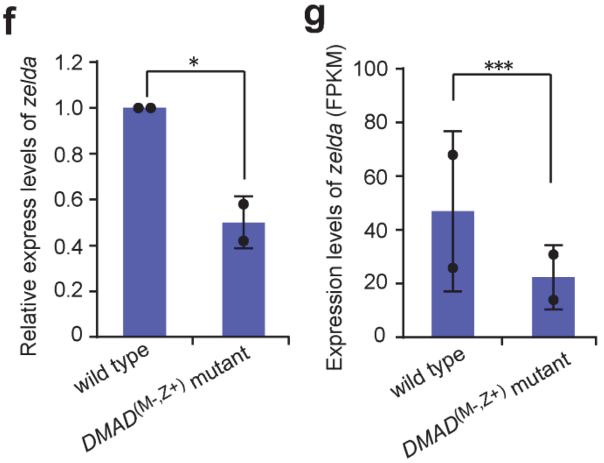

Supplementary Figure 5. Jumu regulates embryonic gene expression via 6mA modification.

(a) The top 20 enrichment gene ontology (GO) terms for genes significantly regulated by the maternal Jumu (GSRJ), Zelda target genes and their overlapped genes. The *P* values of the GO terms were used to produce the heatmap. **(b)** Examples for 6mA and Jumu-binding signals around important genes, such as *lola* and *Trf2*. The enriched motifs in Jumu-bound regions are shown in figures. **(c)** The expression level of *zelda* in wild type, $jumu^{(M-, Z+)}$ mutant embryos at 3-hour stage. Data expressed as means of 2 independent experiments. Error bars indicate mean ± s.e.m. ***, q value from cuffdiff < 0.001. **(d)** Maternal *DMAD* mutation causes partial embryonic lethal. Data expressed as means of 3 independent experiments. Error bars indicate mean ± s.d. The two-tailed Student's t test was used to analyze statistical variance. ***, $P < 0.001$. **(e)** Statistics of hatching rate of embryos with indicated genotypes. *, Sample size. **(f)** Quantitative RT-PCR experiments were used to measure expression levels of *zelda* in wild type and maternal *DMAD* mutant embryos at 3-hour stage. Data expressed as means of 2 independent experiments. Error bars indicate mean ± s.d. The two-tailed Student's t test was used to analyze statistical variance. *, $P < 0.05$. **(g)** The expression level of *zelda* in wild type, $DMAD^{(M-, Z+)}$ mutant embryos at 3-hour stage. Data expressed as means of 2 independent experiments. Error bars indicate mean ± s.e.m. ***, q value from cuffdiff < 0.001. Source data are provided as a Source Data file.

Supplementary Figure 6

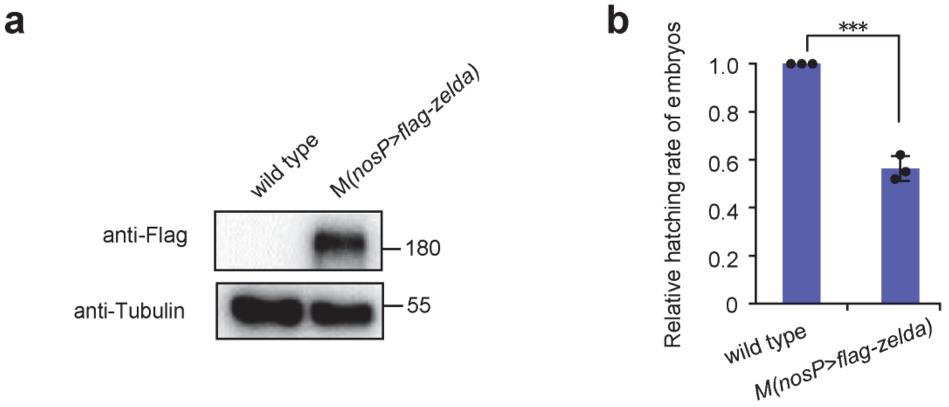

c

Genotype of embryos	Hatching rate		
	Replicate 1	Replicate 2	Replicate 3
Wild type	0.94 (470/500*)	0.93 (352/377*)	0.90 (273/304*)
M (*nosP>flag-zelda*)	0.48 (225/465*)	0.51 (260/505*)	0.56 (225/404*)

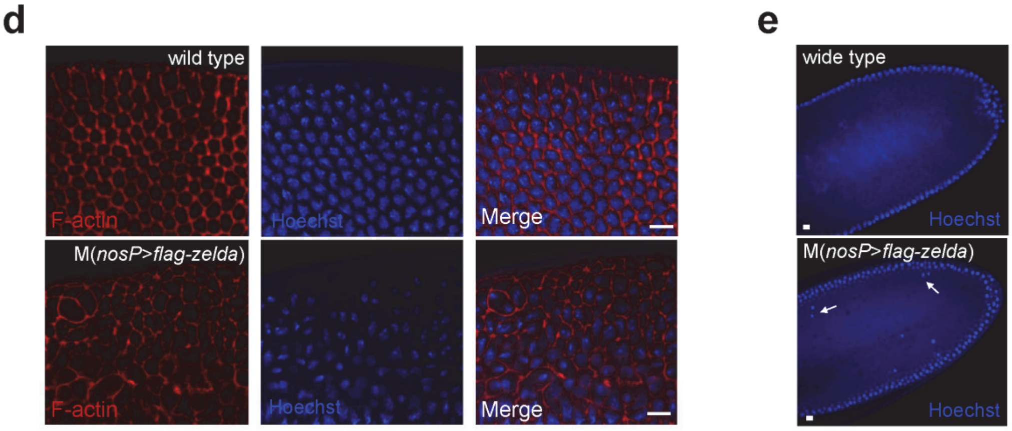

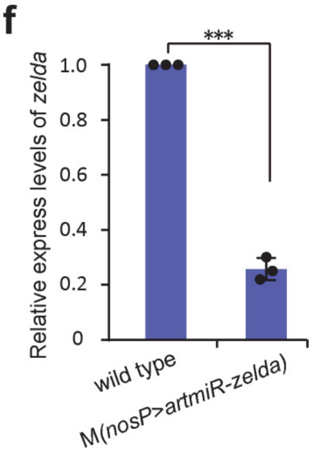

g

Genotype of embryos	Hatching rate		
	Replicate 1	Replicate 2	Replicate 3
Wild type	0.84 (211/251*)	0.89 (279/314*)	0.86 (310/360*)
M(nosP>artmiR-zelda)	0 (0/324*)	0 (0/268*)	0 (0/410*)

Supplementary Figure 6. Maternal Zelda overexpression causes partial embryonic lethal.

(a) Western blot assays were performed to show the expression of Flag-Zelda protein in wild type and maternal overexpressed Zelda embryos. **(b)** Overexpression of Zelda causes partial embryonic lethal. Data expressed as means of 3 independent experiments. Error bars indicate mean ± s.d. The two-tailed Student's t test was used to analyze statistical variance. ***, $P < 0.001$. **(c)** Statistics of hatching rate of embryos with indicated genotypes. *, Sample size. **(d)** The embryos with maternal Zelda overexpression were stained with phalloidin and hoechst to visualize F-actin (red) and DNA (blue), respectively. Scale bar, 10 μm. **(e)** Nuclear fallout in wild type and maternal Zelda overexpression embryos at stage of 9-13 cycles. Scale bar, 10 μm. **(f)** Quantitative RT-PCR experiments were used to analyze the effectiveness of *zelda* knockdown in 0.75-hour stage embryos. Data expressed as means of 3 independent experiments. Error bars indicate mean ± s.d. The two-tailed Student's t test was used to analyze statistical variance. ***, $P < 0.001$. **(g)** Statistics of hatching rate of embryos with indicated genotypes. *, Sample size. Source data are provided as a Source Data file.

Supplementary Figure 7

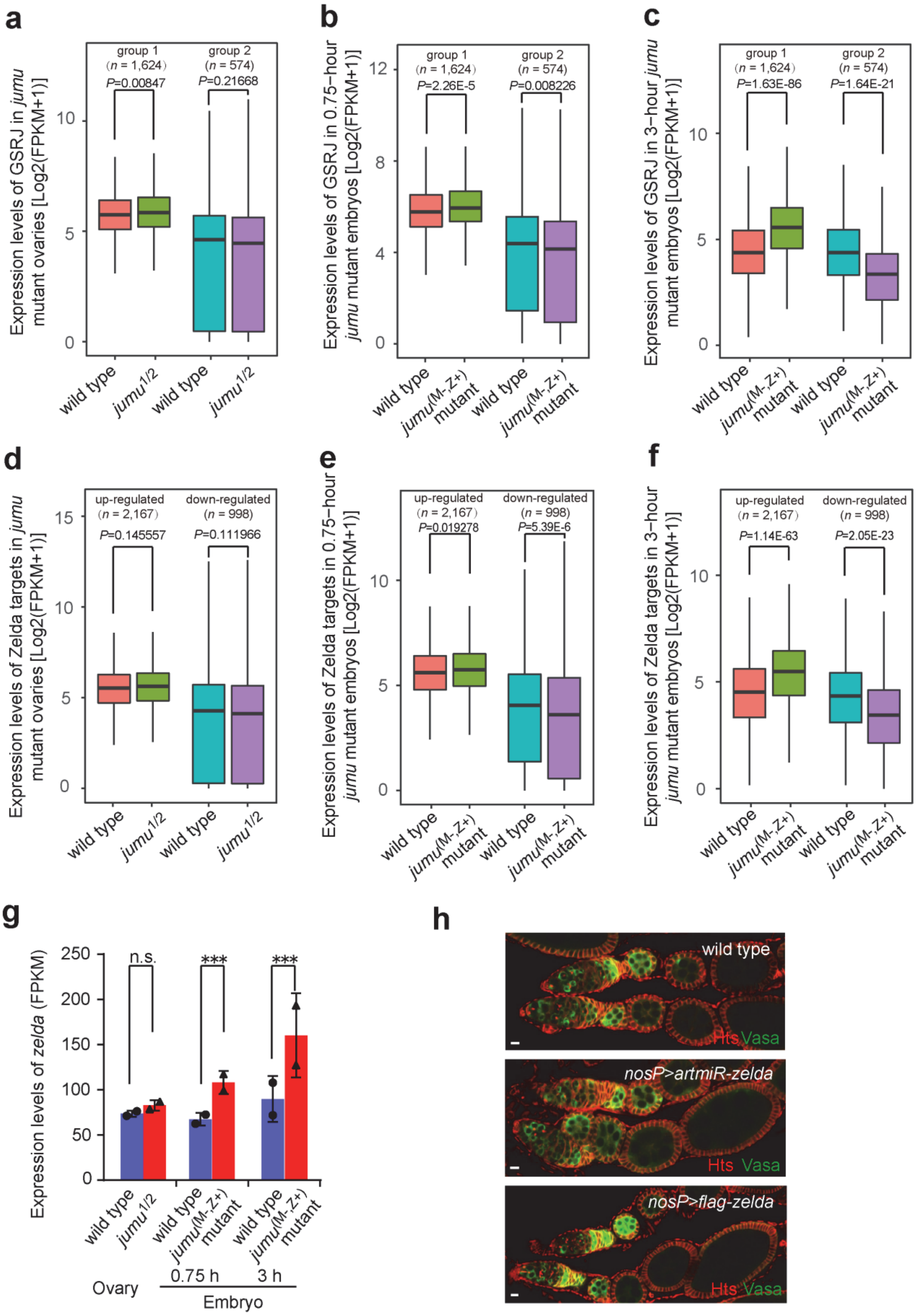

Supplementary Figure 7. Jumu has distinct roles in regulating germline development and early embryogenesis.

(a) Expression levels of group 1 and group 2 genes of GSRJ in wild type and *jumu* mutant ovaries. Loss of Jumu led to significant changes (P=0.00847) of levels of the group 1 genes, although the group 2 genes showed no significant (P=0.21668) change between wild type and *jumu* mutant ovary samples. P values were calculated by one-tailed Student's t test. GSRJ, genes significantly regulated by the maternal Jumu. **(b-c)** Expression levels of group 1 and group 2 genes of GSRJ in wild type and maternal *jumu* mutant embryos at 0.75-hour stage (b) and 3-hour stage (c). Target genes of both group 1 and group 2 genes of GSRJ were more significantly changed at the 3h-stage than these in the 0.75h-stage. P values were calculated by one-tailed Student's t test. **(d)** Expression levels of Zelda target in wild type and *jumu* mutant ovaries. There is no significant change in levels of Zelda target genes (in both groups) between wild type and *jumu* mutant ovaries. P values were calculated by one-tailed Student's t test. **(e-f)** Expression of Zelda target in wild type and maternal *jumu* mutant embryos at 0.75-hour stage (e) and 3-hour stage (f). Zelda target genes were more significantly changed at the 3-hour stage than these in the 0.75-hour stage. P values were calculated by one-tailed Student's t test. **(g)** Expression levels of *zelda* in *jumu* mutant ovaries and maternal *jumu* mutant embryos at 0.75-, 3-hour stages. Data expressed as means of 2 independent experiments. Error bars indicate mean ± s.e.m. ***, q value from cuffdiff < 0.001. n.s., not significant. **(h)** Ovaries from wild type， P{*nos-gvp*}^{3ed} /P{*uasp-artmiR-zelda*} and P{*nos-gvp*}^{3ed} /P{*uasp-flag:zelda*} transgenic flies were stained with anti-Hts (Red) and anti-Vasa (Green) antibodies. Scale bar. 10 mm. For both boxplots, the centre line indicates the median, the bottom and top of the box show the first and third quartiles of the data, and the whiskers show the minimum and maximum values. Source data are provided as a Source Data file.

Supplementary Table 1. The list of primers used in this study.

Primer name	Primer sequence 5'-3'
Jumu target1	TAATACGACTCACTATAGGGAACTAGAGGATTATTCGAG GTTTTAGAGCTAGAAATAGC
Screen-F1	AAGTGCAGAGATCACGGC
Screen-R1	GCGAATTGGCGAGTCAAC
Jumu target2	TAATACGACTCACTATAGGCGGATATAGCTTCCAGTGTT GTTTTAGAGCTAGAAATAGC
Screen-F2	CAATATCAACTGTATTTACA
Screen-R2	TGTGCTGATCCCTTTCGGCG
Reverse	AGCACCGACTCGGTGCCACT
Zelda-KD-s	CTAGCAGTCGAGCACCACTAATCACAAGATAGTTATATT CAAGCATATGTTGTGATTTGTGGTGCTCGGCG
Zelda-KD-as	AATTCGCCGAGCACCACAAATCACAACATATGCTTGAAT ATAACTATCTTGTGATTAGTGGTGCTCGACTG
Zelda-s	GACAAACGCTTCACCACAG
Zelda-as	TGTTGTTTCGTTCAGCAGG
Actin 5C-s	CTGATGAAGATCCTGACCGA
Actin 5C-as	ATGATGGAGTTGTAGGTGGT
DMAD-T7-s	GTGGGTCTGCAAAGTGTGGC
DMAD-T7-as	GCGGATCCTTGGGATCGCTC
gfp-T7-s	ATGGTGAGCAAGGGCGAGGA
gfp-T7-as	CTTGTACAGCTCGTCCATGC
